# Computational Models Using Multiple Machine Learning Algorithms for Predicting Drug Hepatotoxicity with the DILIrank Dataset

**DOI:** 10.3390/ijms21062114

**Published:** 2020-03-19

**Authors:** Robert Ancuceanu, Marilena Viorica Hovanet, Adriana Iuliana Anghel, Florentina Furtunescu, Monica Neagu, Carolina Constantin, Mihaela Dinu

**Affiliations:** 1 Faculty of Pharmacy, Carol Davila University of Medicine and Pharmacy, 020956 Bucharest, Romania; robert.ancuceanu@umfcd.ro (R.A.); adriana.anghel@umfcd.ro (A.I.A.); mihaela.dinu@umfcd.ro (M.D.); 2 Faculty of Medicine, Carol Davila University of Medicine and Pharmacy, 020956 Bucharest, Romania; florentina.furtunescu@umfcd.ro; 3 Immunology Laboratory, Victor Babes National Institute of Pathology, 050096 Bucharest, Romania; neagu.monica@gmail.com (M.N.); caroconstantin@gmail.com (C.C.); 4 Department of Pathology, Colentina University Hospital, 020125 Bucharest, Romania; 5 Faculty of Biology, University of Bucharest, 76201 Bucharest, Romania

**Keywords:** DILIrank, DILI, drug hepatotoxicity, QSAR, nested cross-validation, virtual screening, in silico

## Abstract

Drug-induced liver injury (DILI) remains one of the challenges in the safety profile of both authorized and candidate drugs, and predicting hepatotoxicity from the chemical structure of a substance remains a task worth pursuing. Such an approach is coherent with the current tendency for replacing non-clinical tests with in vitro or in silico alternatives. In 2016, a group of researchers from the FDA published an improved annotated list of drugs with respect to their DILI risk, constituting “the largest reference drug list ranked by the risk for developing drug-induced liver injury in humans” (DILIrank). This paper is one of the few attempting to predict liver toxicity using the DILIrank dataset. Molecular descriptors were computed with the Dragon 7.0 software, and a variety of feature selection and machine learning algorithms were implemented in the R computing environment. Nested (double) cross-validation was used to externally validate the models selected. A total of 78 models with reasonable performance were selected and stacked through several approaches, including the building of multiple meta-models. The performance of the stacked models was slightly superior to other models published. The models were applied in a virtual screening exercise on over 100,000 compounds from the ZINC database and about 20% of them were predicted to be non-hepatotoxic.

## 1. Introduction

Drug-induced liver injury (DILI) has been stated as the most common single cause of drug withdrawal or major regulatory action regarding a medicinal product (such as a labeling change, “black box” warning, etc.) [1,2]. More than 1100 products used by human beings on a relatively frequent basis, such as medicines, herbal and other natural products, minerals, “recreational” or illicit chemical substances have been identified as potentially causing liver injuries; the frequency for some of these is low or very low, however [3]. The clinical image may be varied, from an increase in the level of liver enzymes to hepatitis, cholestasis or liver cirrhosis, and the diagnosis may be very challenging [4]. Two distinct instances of DILI occurrence have been described: one is direct and intrinsic, for which the risk increases proportionally with the dose (e.g., paracetamol) and one idiosyncratic, which only affects susceptible individuals, is not dose-dependent and is consequently not predictable [5] (e.g., nonsteroidal anti-inflammatory agents [6]). Because of the important impact that DILI may have on patient life, as well as of the regulatory impact it has on a drug, early detection of DILI remains a key concern across all phases of the pharmaceutical development and substantial efforts are geared towards this goal [7].

The attempts to predict hepatotoxicity based on cell culture tests are prone to failure, because many compounds that in vivo exert liver toxicity do not kill hepatocytes in vitro or, if they do, they cause their death only at unrealistically high concentrations [8]. This is related to the variability in gene expression of hepatocyte cell lines [8]. Non-clinical studies performed in animals also have limitations that preclude certainty about their ability to predict liver toxicity in humans. The majority of compounds causing idiosyncratic liver injuries in humans could not be detected as doing so in toxicology studies required by the regulatory framework for new drugs [9]. Current computational methods not only have the potential to provide similar performance to the cell culture or animal methods, but they are considerably cheaper, faster and circumvent ethical issues related to animal models. Moreover, using a computational approach coheres with the current tendency for replacing non-clinical tests with in vitro or in silico alternatives, mandated by the implementation of the “3R” principle [10]. This approach is actively encouraged by public authorities such as the European Chemicals Agency (ECHA) or international organizations such as the Organisation for Economic Co-operation and Development (OECD) [11]. Furthermore, computational models allow rapid prediction of the activity of a large number of substances in virtual screening exercises. This is a feat that even with the most sophisticated and automated high-throughput technologies is simply only partially feasible, and at huge costs, considering the expensive targets and ligands necessary [12].

Although the number of computational models attempting to predict DILI published up to now is impressive, many were not based on a reference drug list, and developing such a reference list is a daunting task. In the absence of a “gold standard” defining the DILI risk, the different schema and data sources used to compile previously published annotations has been declared by the FDA researchers as being “of concern” [13]. A first annotated dataset on DILI originated at the FDA and was limited to a small number of 287 active substances; more recently (2016), a group of researchers from the FDA published an improved annotated list of medicines with respect to their DILI risk, constituting “the largest reference drug list ranked by the risk for developing drug-induced liver injury in humans” (DILIrank) [13]. Few Quantitative Structure–Activity Relationship (QSAR) studies have focused exclusively on the DILIrank dataset up to date [14]; published studies have either used other datasets [15], only a subset of the DILIrank [16], or have pooled DILIrank with other data sources [17]. This latter approach may have advantages (increasing the dataset and allowing the development of more robust models), but it also has shortcomings (misclassification bias due to different criteria in annotating drugs from different lists). We have developed a number of QSAR models utilizing a variety of descriptors and machine learning algorithms, and have assembled them to increase the performance. While the majority of the models published to date use only conventional cross-validation, we assessed the performance of our models with the state-of-the-art method of nested (double) cross-validation, which ensures better performance and control of overfitting [18]. Finally, we applied the models to virtually screen over 100,000 compounds of the ZINC 15 database [19] (the compounds with a name in ZINC) and examined bottom compounds (with the lowest probability of being hepatotoxic) to explore the validity of the models on unknown chemical compounds.

## 2. Results

### 2.1. Dataset Analysis

**Figure 1:**
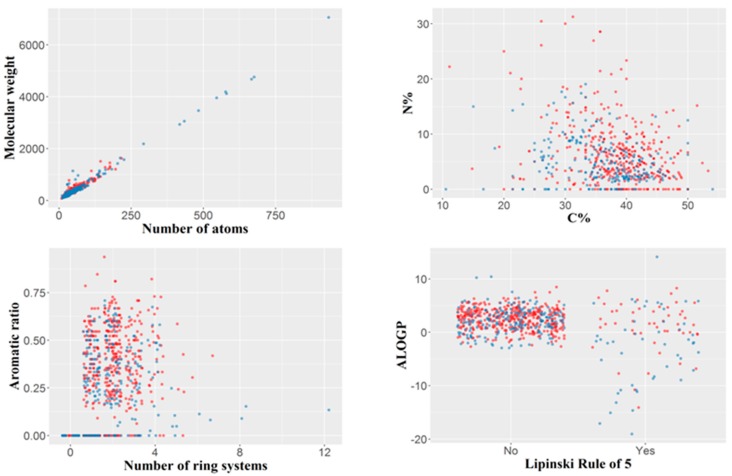
Variability of the dataset illustrated by several simple constitutional descriptors or molecular properties. Blue: compounds of no concern; red: compounds of hepatotoxicity concern. For the Lipinski rule of five, “No” indicates the compounds with no violation of the rule, and “Yes” those violating the rule.

The final dataset included 694 organic molecules with a molecular weight varying from 76 (hydroxyurea) to 7055 Da (ecallantide), with a median value of 329.5 Da and 75% of the molecular weight values less than 430 Da. The number of atoms per molecule varied between nine and 934, the median being 43. The number of ring systems per molecule ranged between zero (aliphatic molecules) and a maximum of 12 (with a median of 2). Only 99 out of the 694 molecules, violated Lipinsky’s rule of five, divided roughly equally between those of concern and those of no concern (50 and 49, respectively). Compounds with molecular weight higher than 2000 tended to be non-toxic, whereas compounds with a nitrogen percentage higher than 20% tended to be hepatotoxic; however, these observations are derived from a relatively small number of compounds. The variability of the dataset by several simple constitutional descriptors or molecular properties is illustrated in Figure 1.

**Figure 2:**
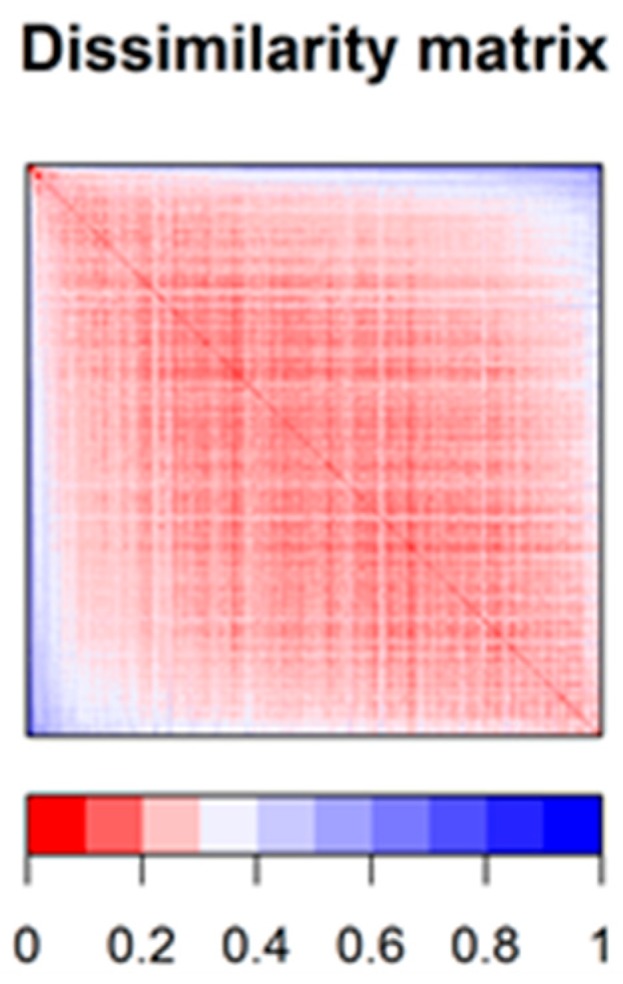
Dissimilarity matrix (based on Gower distance) offering a synthetic image of the chemical diversity in the dataset.

To estimate the chemical diversity in the 694 drugs constituting the dataset, a dissimilarity-based matrix was computed using the rescaled Gower distance [20] (this metric being appropriate for a combination of continuous and binary data). For this purpose, we used the 708 most relevant descriptors (obtained by removing auto-correlated and quasi-constant features) (Figure 2). Most compounds have other constituents from the same dataset that they resemble (scaled distances under 0.25), but also that they are quite unlike other compounds from the same dataset (scaled distances larger than 0.55) (Supplementary Figures S1–S3). The majority of median scaled distance values were about 0.2–0.3, suggesting that the chemical diversity in the dataset was somewhat limited (but since all the substances in the dataset are approved drugs, this should not be very surprising).

### 2.2. Performances of Models

**Figure 3:**
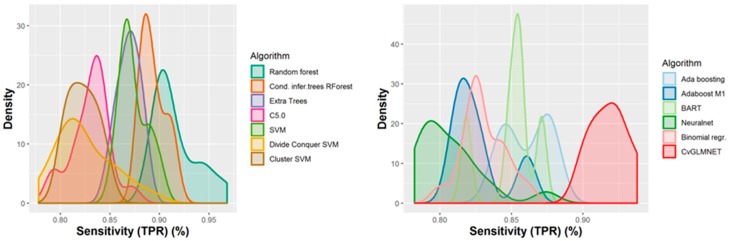
Performance of 165 Quantitative Structure–Activity Relationship (QSAR) models in terms of sensitivity.

**Figure 4:**
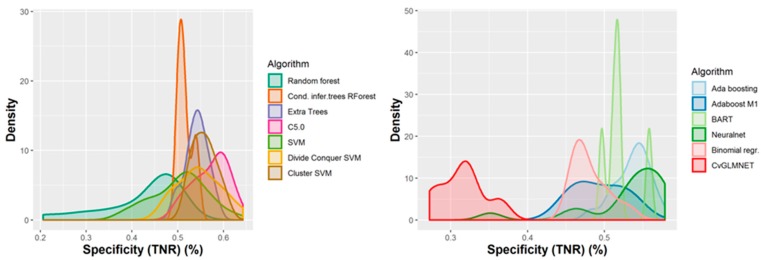
Performance of 165 QSAR models in terms of specificity.

**Figure 5:**
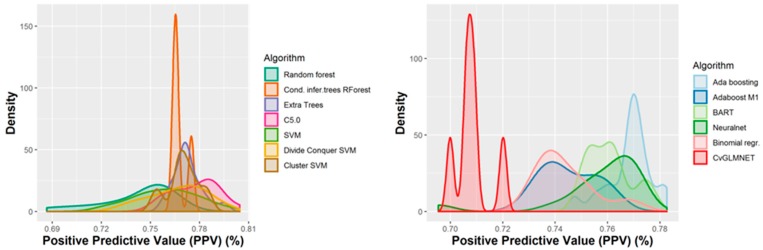
Performance of the 165 QSAR models in terms of positive predictive value.

**Figure 6:**
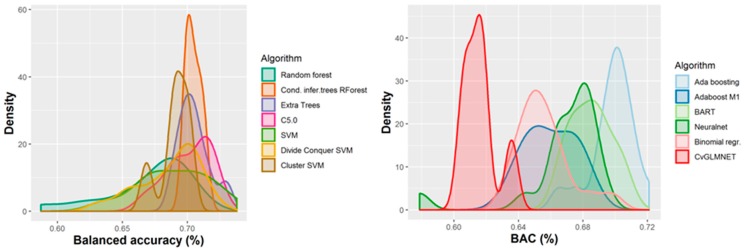
Performance of 165 QSAR models in terms of balanced accuracy.

A number of 267 different QSAR models were built, with different feature selection algorithms and machine learning techniques, of which 79 were selected for assembling by stacking. The performance of the majority of algorithms (165 models, each using 50 features for classification) in terms of sensitivity, specificity and positive predictive values [21], in nested cross-validation, is shown in Figure 3, Figure 4, Figure 5 and Figure 6. Whereas certain algorithms (logistic regression, gradient boosting machine) did not manage to model well the hepatotoxicity, many of the algorithms attempted were useful in building models with a reasonably good performance.

The majority of those models had good or very good performance in terms of sensitivity, which was in most cases over 80% and up to 95%. This good sensitivity, however, came at a cost in specificity, which varied mostly around 50% and in best cases reached or slightly exceeded 60%. This means that about half of the non-hepatotoxic substances are likely to be predicted as hepatotoxic, although they are not so. However, we preferred to sacrifice, to a certain extent, specificity for sensitivity, because our purpose was not to mislabel a hepatotoxic substance as innocuous, rather than the alternative. In this case there is considerably high confidence that a substance is not hepatotoxic if predicted not to be so. The positive predictive value (PPV) was reasonably good, for most models it was around 77%. The balanced accuracy (BA) was also reasonably good, with 79 models (those selected for stacking and prediction use) having a BA higher than 70%. In only six models was BA higher than 72% and, in all cases, it was lower than 73%.

We first assembled the best models through a simple majority vote of binary predictions; this ensured a balanced accuracy of 72.8%, a sensitivity of 89.0% and a specificity of 56.5%. Assembling the models based on the mean probability of all models and a decision threshold of 50% resulted in a balanced accuracy of 72.2%, a sensitivity of 88.3%, and a specificity of 56.1%. Using the same 50% threshold and median probability values slightly improved the performance, but it was not better than that based on the majority vote (balanced accuracy 72.6%, sensitivity 88.8%, and specificity 56.5%). Changing the probability threshold value to 0.67 (instead of 0.5) and using median predicted probabilities leads to the best performance in terms of balanced accuracy (74.6%), with a lower sensitivity (76.0%) and improved specificity (73.2%).

The two best-performing meta-models built by applying the random forest classifier to the binary predictions of 50 models (selected by applying the same feature selection algorithms) and the maximum daily dose, had a balanced accuracy of 74.38% and 74.20%, respectively. These two meta-models had a high sensitivity (89.68% and 89.71%) and, thus, low false negative rates (10.32% and 10.29%). They are, therefore, particularly useful to ascertain whether an unknown compound is devoid of liver toxicity properties. Assessing each of these meta-models with different random seed numbers slightly decreased the performance for one of them and increased it for the other (mean balanced accuracies for five repeated runs with different seed numbers were 73.56% and 74.27%, respectively; standard deviations 0.47% and 0.44%). The inclusion of dose among the predictors in the meta-models only slightly (if at all) increased the performance compared with the meta-models built without the dose, but we preferred to include it on the basis of domain knowledge [22,23]. Meta-models built similarly with support vector machines (SVM), k-nearest neighbors (knn and its Rweka implementation, IBk) and naïve Bayes algorithms had a slightly lower performance in terms of both balanced accuracy and sensitivity than those built with random forests.

Using the predicted probabilities to build meta-models with random forests in a similar way with 50 models and the maximum daily dose as features did not improve the performance in terms of both balanced accuracy and sensitivity. Instead, the computing time increased by about five times. Meta-models built with naïve Bayes on output probabilities had the highest balanced accuracy (mean of five runs with different seeds 74.64%, standard deviation 0.20%), but a lower sensitivity (83.0%) and higher specificity (66.3%). Using other algorithms (knn, IBk, SVM, ksvm, C5.0) for the construction of meta-models had very similar results, with resembling performances for each selection algorithm (70.69–74.83% balanced accuracy, 82.81–90.32% sensitivity, 54.90–64.47% specificity). Adding the typical duration of treatment (in days) as an additional feature had a minimal effect on the meta-model performance. The number of days up to the first occurrence of liver toxicity might be more relevant, but we could not collect data for each drug substance in the dataset for this variable. The feature selection algorithms applied to the 72 models outputting the probabilities identified as the most important for prediction in the following classification algorithms: ksvm, SVM, Adabag Boosting, kknn, IBk, random forest based on conditional inference trees, and ada. The most efficient feature selection algorithms thus identified were the OneR association rule and the “randomForestSRC_var.select” of the randomForestSRC package [24].

### 2.3. y-Randomization Test

The y-randomization test showed that in all cases the performance was considerably worse after the scrambling of the response variable. In all cases the balanced accuracy was close to 50% in the case of the scrambled datasets, whereas it was generally over 70% in the case of the genuine models (Figure S4). The other parameters were also considerably worse when compared with the genuine models: the AUC was close to 50%, whereas in many cases the true positive rate (TPR) and false positive rate (FPR) were 100% (all compounds were classified into a single class). The repeated similar performance of the randomized datasets for different classification algorithms and feature selection algorithms confirms that our models are not the result of mere chance. On the contrary, they seem to reflect a genuine relationship between the chemical structure as measured through the molecular descriptors used and the degree of DILI concern.

### 2.4. Descriptors Associated with Hepatotoxicity

Although multiple QSAR models have been developed for DILI substances, often the articles published were focused more on the performance of the models than on the discussion of the descriptors that are associated with an increased or lower risk of liver toxicity. In order to identify the most important features/descriptors associated with DILI, we examined the first five descriptors identified by each selection algorithm. We, therefore, computed the frequency with which descriptors occurred among the most important five features for each of the selection algorithms and those that occurred at least twice are shown in Table 1. A higher mean atomic polarizability tended to be associated with a higher DILI concern; similarly, a lower percentage of hydrogen in the molecule and a lower Geary autocorrelation of lag 1 weighted by mass tended to be associated with a higher risk of hepatotoxicity.

### 2.5. Virtual Screening

Besides using the nested cross-validation procedure, which offers considerably stronger safeguards than merely using an external hold-out sample for external validation, we would have liked to test our stacked models on an independent external dataset. For reasons shown in the Discussion section, this was an almost impossible mission; we therefore chose to apply the models in a virtual screening exercise on 104,619 compounds from the ZINC database, so as to identify compounds having a high probability of being devoid of liver toxicity properties. 19.92% of the whole dataset (20,835 substances) were predicted by the assembly of 72 models based on the mean probability to be non-hepatotoxic and 20.08% (21,012 substances) were predicted by the 72 models based on the median probability to be non-hepatotoxic. The false negative rate of the stacked models, using the average probability of hepatotoxicity for the 72 models was 11.7%, whereas the negative predictive value was 72.6%, which means that if the same proportions between hepatotoxic and non-hepatotoxic compounds was present in the tested dataset, one should expect that 72.63% of the compounds predicted to be non-toxic should indeed be non-toxic. We show in Table S1 the first 2000 such compounds predicted to be devoid of liver toxicity, sorted by the predicted probability (i.e., the first 2000 compounds with the lowest probabilities of being hepatotoxic). As discussed in the next section, all or almost all of these 2000 compounds were in the applicability domain for at least a fraction of the models used. A short look over these compounds shows that they include many oses, polyols, short peptides, vitamins, various hydrosoluble compounds, and this makes credible these predictions, at least for the majority of the compounds.

### 2.6. Outliers, Applicability Domain and Wrongly Classified Drugs

An outlier may be defined as “an observation in a dataset which appears to be inconsistent with the remainder of that set of data” [25]. Outliers may lead to wrongly specified models and wrong results, but they may also be carriers of important information [25]; therefore, a decision to remove them should be based on well-founded reasons (e.g., obvious recording error), and not on the mere intention of having well-performing models. A variety of model-dependent (parametric) and model-independent (non-parametric) methods have been advanced in the literature. Nevertheless, the comparative performance is difficult to estimate, and different benchmarking studies compared different algorithms, sometimes with inconsistent results [26,27]. Because in one complex benchmarking study [26] the isolation forest (IFOREST) and subspace outlier detection (SOD) algorithms were among the best (IFOREST had the highest performance), we used the two algorithms to examine the potential outliers and how well they were predicted by the models. The IFOREST algorithm identified no obvious outlier (for this algorithm, potential outliers have scores close to one, whereas, for all observations of the dataset, the scores varied between 0.30 and 0.55). For the SOD algorithm we used a 5% threshold for the definition of outliers (thus identifying 35 outliers), and over three quarters of them (78.49% on average, s.d. 5.16%) were correctly classified by the assembly of models selected.

The “applicability domain” (AD) is a tool used to assess whether a QSAR model may be employed to predict, in a valid manner, the class label of a test compound; such a “prediction” is only valid if the assumptions on which the model was built are still met for the test compound [28]. If the prediction exercise involves an extrapolation from the feature space, the result of this exercise cannot be relied upon. Hence, evaluating the AD for a specific model is of key significance if that model is to be put to use for prediction purposes. A great variety of methods have been advanced in the literature in relation to this, each with its own strengths and shortcomings [29]. Our approach of normalization and capping extreme values to two standard deviations was meant to ensure a broad AD for untested substances and we assessed this on a subset consisting of the first 2000 substances from the ZINC database, for which there is a high probability that they are not hepatotoxic (the first 2000 compounds with the lowest computed probabilities of being hepatotoxic). The method of F. Sahigara et al. (2013), which uses different decision thresholds for each test compound, identified only a small number of compounds that were out of AD, and only for a subset of the models. The application of this method, therefore, led only to small changes in the average probability predicted for each compound, with no change in the predicted label. The same was true for the use of the Influenced Outlierness (INFLO) and Connectivity-Based Outlier Factor (COF) methods. The INFLO algorithm compares the density of an observation of interest with the average density of its neighboring data points [30]. The Connectivity-Based Outlier Factor (COF) algorithm is based on a distinction between “low density” and “isolativity”, and uses so-called “chaining distances” to compute a COF that shows how much an observation deviates from a (local) pattern [31]. Although leaving out the models that were outside of the AD did not change the classification of any of the 2000 compounds analyzed, in some cases, particularly in the case of using the COF method, leaving aside some of the models (that were outside AD) tended to slightly increase the average probability of a compound being hepatotoxic, but in no case did it reach 50% so as to change the classification.

We analyzed the hepatotoxic compounds from the DILIrank dataset that were wrongly classified by the majority of models and identified a number of 49 such substances out of 447 compounds with different levels of hepatotoxicity concern (i.e., 10.96% false negative rate). Among these, ethambutol was predicted wrongly by all models (as non-toxic, although it is labeled as of “most concern”); in other words, all models predict ethambutol as non-hepatotoxic, although it has been categorized in the DILI Rank dataset as being of high concern. This is in line with some medical papers that consider ethambutol as non-toxic [32]. According to LiverTox, despite over a half of century of use, ethambutol has been connected to clinically evident liver injury in a very small number of case reports, but some of these were convincing (re-occurrence by rechallenge) [33]. This prompts us to hypothesize that such rare cases of confirmed hepatotoxicity might have been related to an impurity present in the active substance or one of the excipients, although it may as well be possible that the models were just unable to classify it correctly. Perhexiline, daptomycin, and amphetamine, were correctly classified by a single model out of 79, terbutaline by two models, and acarbose by only three. All other compounds misclassified by the majority of the models are shown in Table S2. Only 14 of these 49 compounds (28.57%) were detected as outliers for at least one of the models, a fact that indicates that, in the majority of cases, the wrong classification is not the consequence of different chemical features, but rather of the model’s limitations.

## 3. Discussion

Predicting hepatotoxicity of chemical substances from their chemical structure is an intimidating task, because the mechanisms by which different substances cause liver toxicity may be varied and they are often not understood at all. Moreover, the hepatotoxic substances differ in their liver toxicity profile with respect to the doses or duration needed for the toxic effect to be manifested, as well as in the clinical severity of the hepatotoxicity (varying from slight increases in transaminase levels to fulminant hepatitis requiring an emergency liver transplant). Dactinomycin, for instance, may manifest its liver toxicity at total doses of about 1 mg/day (10 to 15 mcg/kg) [34], whereas 4-aminosalicylic acid has been used at daily doses 12,000 times higher [35]. Hepatic injury may be started by paracetamol from 24 to 72 h after a single overdose [36], in the case of allopurinol hepatitis, injury usually develops within the first month of treatment, whereas clinically symptomatic liver injury is seen with perhexilin only after several months or years of treatment [37]. Drug-induced hepatotoxicity may be predictable (dose-dependent, replicable in animal experiments, and with a short onset), but, in most cases, it is idiosyncratic (non-predictable) [38]. The latter is often classified in three main mechanistic patterns: hepatocellular, cholestatic, and mixed [39]. Other mechanistic classifications are considerably more nuanced, distinguishing between immune-mediated and non-immune-mediated hepatitis, non-alcoholic steatohepatitis, immune- and non-immune-mediated cholestatic injury, fibrosis/cirrhosis, granulomas (allergic in nature), microvesicular steatosis, vascular lesions, phospholipidosis, and neoplasms [40]. Considering this diversity of doses, latencies and mechanisms, the chemical diversity of the drugs currently used in therapy, as well as their limited number, predicting hepatotoxicity from the structure alone is fraught with difficulties.

Multiple studies have concluded that the agreement between the results of animal liver toxicity models and human outcomes is low, and this may contribute to a high number of cases where the hepatotoxic potential of a drug is only detected in the late stages of clinical development [41]. To further complicate modeling, classifying a known drug as being hepatotoxic or not is not a simple exercise, because for the majority of medicines the liver toxicity is idiosyncratic and if the toxic events are relatively rare, they cannot be detected in clinical trials, but only through post-marketing studies, helped by the spontaneous reporting pharmacovigilance systems, which themselves tend to suffer from underreporting [13]. Taking these factors into account, as well as the diversity of clinical forms of DILI and the ambiguities affecting the causality assessment, the fact that data from animal studies may not necessarily be relevant for the classification of a drug as hepatotoxic or not, different sources may classify differently specific drugs (i.e., the same drug may be considered hepatotoxic in one study/list, but non-hepatotoxic in another). For instance, leuprolide has been classified as of “Less DILI concern” in DILIrank, whereas the LiverTox website (produced by the National Institute of Diabetes and Digestive and Kidney Diseases (NIDDK)) states that “Despite use for several decades, leuprolide has not been linked to convincing cases of clinically apparent liver injury. Routine monitoring of patients for liver test abnormalities is not recommended. Likelihood score: E (unlikely cause of clinically apparent liver injury)” [42]. Morphine and codeine are listed by DILIrank as of no DILI concern, whereas a recently published list of (mostly herbal) ingredients with potential hepatotoxicity or hepatoprotection, labels both of them as a hepatotoxic [43]. The same list labels lamivudine and metformin as hepatoprotective [43], whereas DILIrank classifies them as hepatotoxic (of less concern).

The DILIrank dataset is based on a “refined annotation schema by weighing evidence of causality to overcome inherent deficits in drug labeling and improve the accuracy of DILI annotation” [13]. When we finished the modeling exercise, it was still the best publicly available annotated list of drugs, classified by their hepatotoxic potential, based on clinical considerations. When we were in the final stages of drafting this paper, an improved version with a larger number of drugs was just published by the same group of FDA-affiliated authors, under the name DILIst [41].

Predicting liver toxicity from chemical structure has been a preoccupation of over two decades, first starting somewhat timidly with local models [44,45], to move later also to global models [46,47]. Many of the previous models used relatively small size datasets (less than 400 compounds) [48,49]; studies based on datasets larger than DILIrank have also been published [11,17,50,51,52], but the authors of DILIrank used a methodology that, in theory at least, was superior and more consistent with the totality of available clinical data. The total DILIrank dataset includes 1036 compounds, but 254 were classified as of “ambiguous DILI-concern”, because the causality evidence was limited; besides, it took great care in defining DILI negatives, which varied among four large sources previously studied, labeling some compounds previously considered of no DILI concern as of less DILI concern. Thus, although somewhat smaller than other published DILI datasets, the DILIrank had a smaller probability of misclassification, and this prompted us to prefer it to other larger datasets available in the literature. 

All or almost all studies published up to date have used a training and a testing dataset, and most often also a holdout (external validation) dataset, in most cases quite a small one. None of the previously published studies have used a nested-cross validation approach. It has been the tradition, in the field of machine learning, to divide the whole dataset in a training subset (about 70–80%) and a test set (about 20–30%); the models were developed, hyperparameters tuned and performance evaluated on the training subset, and the selected model was then tested on the test set (holdout). In this approach, it is still possible to have a model with good performance on the holdout test by mere chance. The nested cross-validation splits the dataset in training and testing sets multiple times, with the best model being tested on an external dataset that has not been contaminated by the training samples each time. The main difference is that whereas in the traditional approach, a single holdout test was kept for assessment, in the nested cross-validation (CV), the process is repeated multiple times (in our case, 10 times). The performance on the training dataset is of little interest, because it is known that the available algorithms can overfit and have apparently very high performances. The performance on the test datasets should also be of little interest, because it is also quite common for the algorithms used to get a good performance on the test set by mere chance. The performance, as measured on external validation datasets, is the most important and it is interesting that, whereas models published up to now have reported good and excellent performances on the training or test subsets, the performance on external datasets was much lower. A review found, in 2014, that in models published up to that time point, the external validation datasets had been “quite small” (20–50 drugs), and that for the larger external datasets the model performance seemed to be “less favorable” [53]. One of the best-performing ensemble models recently published used three external validation datasets and had a pooled balanced accuracy of 71.60% [17]. Although based on a different dataset (DILIrank), our model compares favourably, with a balanced accuracy in the nested cross-validation (i.e., an average of 10 external datasets) that is slightly superior for several meta-models attempted and higher than 74% (as shown in the results section).

We were interested in comparing our results with those of the PROTOX II (http://tox.charite.de/protox_II/index.php?site=compound_input), which is built on a training set of 850 compounds and an external dataset of 95 compounds, but such a comparison proved not to be realistic. Our predictions were all made as part of an external dataset (we used the predictions in the outer test set of the nested cross-validation loop), whereas in the case of PROTOX II, [54], the majority of compounds are likely to be part of the training set, so are not really predictions. Moreover, the labels used in the training set of PROTOX II seem to be very different from those used in DILIrank; there is a large agreement between our results and PROTOX II for the compounds classified as of most concern by DILIrank, yet the large majority of compounds labeled by DILIrank as of less concern, are “predicted” by the PROTOX II to be “inactive”. This is obviously not due to a failure of the PROTOX II in prediction, but rather in the different labels used in its training dataset. Purely for information purposes, we show the results of the PROTOX II in comparison with our predictions in the external loop in Table S2.

Among the most important chemical descriptors associated with liver toxicity in our study were the mean atomic polarizability (Mp), the percentage of hydrogen atoms (H%), the Geary autocorrelation of lag 1 weighted by mass (GATS1m), normalized spectral positive sum from Burden matrix weighted by mass (SpPosA_B(m)), and Moriguchi octanol–water partition coefficient (MLOGP). Atomic polarizability, identified as important by the majority of selection algorithms used, was previously shown to associate with renal toxicity of drugs [55], but it was also used in other liver toxicity models, as was logP [17,56], the latter measuring lipophilicity, which was shown to correlate with DILI [23]. GATS1m is less intuitive than the constitutional descriptors, but it has also been reported in a different publication as an important descriptor for the liver toxicity of drugs [57]. SpPosA_B(m) is also less easily interpretable, and we did not find any previous use in other QSAR models of hepatotoxicity.

Many papers have developed QSAR DILI models, but very few, if any, applied those models to a large number of substances in a virtual screening exercise, so as to estimate the chances of finding substances devoid of hepatotoxicity. Our virtual screening of over 100,000 substances from the ZINC database found that about 20% of the substances from this dataset have a high probability of being devoid of any liver toxicity. Because our models tended to have a relatively high rate of false positives, and the specificity was only about 56%, the proportion of non-hepatotoxic substances is probably much higher in the dataset than 20%. We have examined the list containing the first 2000 such substances, searching for published information about them in PubMed, as well as looking at their chemical structure and hydrophylicity. Such a step tends to confirm the validity of the predictions. Nordihydroisomorphine, the first compound, for instance, is a metabolite of hydromorphone that has been isolated from urine [58]; it seems likely, therefore, that it is hydrophilic and needs little liver metabolizing. It is known that drugs with higher lipophilicity tend to be hepatotoxic, whereas those with low lipophilicity tend to be non-hepatotoxic [59]. Trihexyphenidyl or adrenor (norepinephrine) were already in the dataset as non-hepatotoxic. Calystegine B5 has four –OH groups, exhibiting a relatively high hydrophilicity. Dibekacin, butirosin or fortimicin are aminoglycosides, and in the DILIrank other aminoglycosides were classified as of “no DILI concern”. Dimetipirium and pentolinium are quaternary ammonium derivatives, and other such derivatives were included in the DILIrank list among the harmless compounds (vecuronium, ambenonium, edrophonium etc). Many compounds in the list are small peptides (Lys-Gly, Arg-Gln etc), small sacharides (arabinofuranobiose, digitoxose, arabinose) or polyols (6-deoxy-L-gulitol, dideoxyiminoxylitol, dulcitol, 2-methylerythritol etc). A small number of the compounds predicted to be non-hepatotoxic are, in fact, known to be hepatotoxic, such as cycasin [60] (probably because the models ignored the azoxy moiety, basing the decision only on the multiple hydroxy groups).

The DILIrank dataset is very limited (after excluding non-modelable entities, such as those with ambiguous DILI concern, mixtures or inorganic compounds, only 694 chemical structures), and this leads to small chemical diversity and small learning power for the machine learning algorithms. The very recent publication of the DILIst [41] opens up the possibility of using a larger dataset, which is expected to allow better performance of QSAR models built with its help. Our preliminary data show that including the daily dose and duration of therapy may slightly increase the performance of the models. We intend, in the future, not only to use the larger DILIst dataset, but also to attempt the improvement of the models by using such variables as dose and duration of treatment. This is justified by their influence on the liver toxicity of a product (if paracetamol is used at low doses, it is very unlikely to be hepatotoxic, as it is if perhexilin were to be only used for a few days). Despite the fact that using only chemical descriptors for model building in the case of a defined molecular target makes full sense, in the case of a heterogeneous and more-or-less black box effect, such as liver toxicity, it stands to reason that additional considerations related to the context of use (dose, duration) should also be taken into account and explored in future research.

## 4. Materials and Methods

### 4.1. Dataset

The dataset (Table S1) was downloaded from the FDA website (https://www.fda.gov/science-research/liver-toxicity-knowledge-base-ltkb/drug-induced-liver-injury-rank-dilirank-dataset) and included 1036 drugs, classified in four groups: 192 labeled as “Most-DILI-concern”, 287 “Less-DILI-concern”, 312 ”No-DILI-concern”, and 254 “Ambiguous-DILI-concern”. Because “ambiguous” is not an actual outcome, but rather a category for which there is uncertainty on their DILI-inducing potential, this last category was excluded from our analysis, reducing the size of the dataset to 791. Biological medicines (e.g., abatacept, abciximab, etc.), drugs that are not definite chemical entities (mixtures—e.g., divalproex sodium, which is a coordination compound of sodium valproate and valproic acid, polymers—e.g., polyethylene glycol 3350), and a small number of simple compounds (e.g., “sterile water”, “calcium acetate”, “cisplatin”) were also eliminated. The final dataset consisted of 694 chemical compounds, of which 179 of most DILI concern, 268 of less DILI concern, and 247 of no concern. We collapsed the most DILI concern and less DILI concern in a single category (of DILI concern), so as to apply binary classification algorithms. The corresponding smiles were included in the dataset provided by FDA; we converted them to 2D chemical structures (sdf) using Discovery Studio Visualizer v16.1.0.15350 (Dassault Systèmes BIOVIA, San Diego, CA, USA). We checked the correctness of the resulted formulas with the Chemaxon Structure Checker v. 18.8.0 (ChemAxon, Budapest, Hungary) (no errors found) and used the Chemaxon Standardizer v. 18.8.0 to neutralize, tautomerize, aromatize, and clean 2D the formulas (in this order).

### 4.2. Descriptors

Dragon 7 software (version 7.0, https://chm.kode-solutions.net; Kode SRL, Milano, Italy) was used to compute 3839 molecular descriptors (2D), based on the sdf structures of the chemical compounds in the dataset. A total of 19 blocks of molecular descriptors were computed (Table S3).

### 4.3. Feature Selection

High dimensions of the data (and “high” may refer to billions of observations, but even 691×3891 data points is not exceedingly small), pose a challenge for data analysis. It is likely that—in our case—among the several thousand of descriptors are not all correlated with the activity and there is likely much noise or redundancy. The latter should be removed in order to build parsimonious models that are not overfitted and are useful for prediction purposes [61]. Many if not most machine learning algorithms have actually been developed for a fairly low number of variables, and using a very large number of features will likely result in overfitting [62]. It is, therefore, a requirement to remove noisy and redundant features with the help of one or more feature selection algorithms [63]. A variety of such algorithms have been published, but few comparative performance data with respect to these algorithms are available.

We removed the constant and quasi-constant features (those with less than 1% variation from the statistical mode value) and auto-correlated features (correlation coefficient > 0.9). On the dataset thus reduced we have applied 17 distinct feature selection approaches. Some of these are available through the R “mlr” package [64] directly: “anova.test” (based on ANOVA), “auc” (based on the area under the curve), “kruskal.test” (based on a Kruskal–Wallis test rank sum test), and “permutation.importance” (based on the aggregate difference between predictions performed with the unmodified and permuted features), “univariate.model.score” (based on resampling a recursive partitioning learner with each separate feature), and a simple test based on variance. Others are available through the FSelector R package [65]: “FSelector_chi.squared” (uses a chi-squared test of independence between each variable and the outcome), “FSelector_gain.ratio”, “FSelector_information.gain”, and “FSelector_symmetrical.uncertainty (entropy-based filters), FSelector_oneR (applies the OneR algorithm), “FSelector_relief” (based on the Relief algorithm as updated by Kononenko et al.). Other feature selection algorithms included “party_cforest.importance” (permutation importance implemented in the “party” package [66]), “ranger_permutation” (permutation importance implemented in the “ranger” package [67]), “ranger_impurity” (based on ranger impurity importance), and three selection algorithms based on random forests (implemented in the “randomForest” [68] and “randomForestSRC“ packages [24]). Classification algorithms (discussed below) were applied on different subsets of features selected with these feature selection methods (multiple classification algorithms were applied to each subset of the features thus selected).

Although it is a frequent practice to remove outliers when building QSAR/QSPR models, based on the wrong prediction by the majority of models [17] or other approaches [69], we preferred to eliminate no value. Although this practice (of elimination) might be justified, it may also lead to overestimation of the performance of the models, because the fact that the majority of models are not able to correctly classify an observations does not necessarily imply “outlierness“ for that value. We prefer to identify and discuss outliers but to build models being aware of their presence and the limitations they bring upon the models in terms of performance.

### 4.4. Classification Algorithms

We have applied the following algorithms to build classification models for the DILIrank dataset: binomial regression; regularized regression; C5.0 decision trees and rule-based models; random forests, regularized random forests, and random forests based on conditional inference trees; rotation forests; extremely randomized trees; Bayesian additive regression trees; support vector machines, clustered support vector machines and divided-conquer support vector machines; Ada boosting; regularized and shrinkage discriminant analysis; neural networks (in three different implementations). All algorithms were applied in the computing and programming environment R, v. 3.6.1 [70], under the unified interface provided by the “mlr” R package [64] coupled with “parallelMap” [71] for parallel computing. For data pre-processing, the “caret” package [72] was also used.

Binomial regression (logistic regression), is a fairly simple classification algorithm that models the probability that a certain instance belongs to one of two classes in a linear manner [73]. In essence, logistic regression estimates the probability P = 1/(1+e^−t^), where t = a_0_ + a_1 × 1_ + a_2 × 2_ + … + a_n × n_ [74]. Regularized regression is a slightly more sophisticated form of conventional regression, where the loss function besides minimizing the sum of squares uses a penalty term. Depending on its value, the regularized regression takes three different shapes (variants): ridge regression, lasso regression, and elastic net regression, each with its own strengths and weaknesses [75]. We implemented different forms of regularized regression using the “glmnet” R package [76].

C5.0 decision trees and rule-based models have been first advanced by R. Quinlan in 1992, under the name “C4.5”, which was an extension of a previous algorithm called Iterative Dichotomizer 3 (ID3); C4.5 was later improved into the new C5.0 classifier, which has superior efficiency [77]. C5.0 decision trees are versatile, swift and easy to use, and their use for QSAR modeling is seen as a reasonable option [78]. In R, the C5.0 algorithm is implemented in the C50 R package [79].

Random forests (RF) are a widespread classification algorithm in a variety of fields, including QSAR [80,81]. They assemble a large number of decision trees with the help of a simple majority vote to resolve the most probable class for each data point. The trees are built using random subsets of both the instances from the training set and of the features in building the individual trees [82]. We applied the algorithm as implemented in the “randomForest” R package [68]. Regularized random forests are an adjustment of the conventional random forests geared towards improving the feature selection process by penalizing the introduction of new features in comparison with the previous trees. New features are added only if they provide substantially new information gain/predictive information [83]. It is implemented in R by the original author (H. Deng) in the “RRF” R package [84]. A particular type of tree assembled in random forests is the conditional inference tree, which is developed within a so-called “conditional inference framework”. Briefly, a global test of independence between response and features is applied. If the null hypothesis is rejected, the variable with the strongest association with the response is selected; a binary split is performed in that variable and the process is repeated recursively [85]. It is implemented in R by its author, T. Hothorn, in the R package “party” [66]. Rotation forests are a more sophisticated form of random forests, where feature extraction (e.g., principal component analysis) is applied to subsets of features in an attempt to build “accurate and diverse classifiers” [86]. It is implemented in the R package “rotationForest” [87]. The extremely randomized trees (ERT) algorithm is similar to the RF but tends to lessen the variance of the model by the use of a more pronounced randomization component. It differs from RF in two main aspects: (a) each tree is built with all training samples (instead of random subsets) but (b) each node split is chosen randomly in building each tree, instead of using the best split [88]. ERTs are implemented in the R package “extraTrees” [89].

First proposed by H.A. Chipmann et al. in 2010 [90], the Bayesian additive regression trees (BART) represent a form of flexible Bayesian non-linear regression which has been shown to have a similar level of performance with other machine learning approaches—for instance, with random forests [91]. It is regarded as a successful blend of the advantages of the Bayesian approach with the efficiency of random forests [92]. The BART algorithm was used in the R implementation of the package “bartMachine” [93]. The naïve Bayes classifier uses the Bayes theorem to compute probabilities and assumes the independence of all variables conditioned on the class. This assumption rarely holds true in real life (justifying the “naïve” label), but the performance of the algorithm may be surprisingly good in a wide range of classification tasks [94]. In this paper we have used the naïve Bayes classifier only in the building of meta-models, as implemented in the “e1071” R package [95].

The support vector machines (SVM) algorithm employs a range of kernel functions (e.g., linear, polynomial, radial, etc.) to maximize the decision boundary between classes and to define a hyperplane able to best discriminate the classes [96]. It is an algorithm apt for dealing with a large number of features and has been used with good results to solve a diverse range of classification and regression tasks, including QSAR investigations [97,98]. We used the implementation of the algorithm in the R package “kernlab” [95]. Linear, radial and sigmoid kernels were used. Clustered support vector machines (clusteredSVM) is an algorithm proposed in 2013 by Quanquan Gu and Jiawei Han, as a solution for nonlinear data, with a “considerably lower time complexity than nonlinear classifiers” [99]. Briefly, it first partitions the data in clusters (e.g., by k-means), and in each cluster is trained a linear SVM, and a global regularization is applied to prevent over-fitting. The authors reported superiority over linear SVM and similar or superior performance over kernel SVM with better computational efficiency [99]. Although SVM has been described as “probably the most widely used classifier”, the kernel SVM has difficulties when the sample size becomes very large because of the huge computational costs [100]. The divide-and-conquer SVM (DC-SVM) approach, proposed in 2014 by C.J. Hsie et al. manages to break through the sample size barrier, ensuring faster computation speeds and prediction accuracy superior to the approximate solvers used by the conventional kernel SVM algorithms [100]. We used both clusterSVM and DC-SVM as implemented in the R package “SwarmSVM” [101].

Boosting is a concept developed gradually in the field of machine learning. After Kearns and Vazirani were the first to ask whether a “weak” classifier (one performing only marginally better than random) may be “boosted” into a successful, “strong” classification model, Robert E. Schapire developed the first such working algorithm in 1989, whereas his colleague Yoav Freund proposed a more efficient one in 1990 [102]. Adaboost was put forward by the two researchers in 1995; it iterates the application of a weak (base) algorithm, at each iteration adjusting the weights of the wrongly classified instances, thus “forcing” the algorithm to correctly classify those instances [102]. We applied the algorithm in two implementations, one from the R package “ada” [103], the other from the RWeka package [104].

Regularized discriminant analysis (RDA) is an improvement of the linear discriminant analysis (LDA), the first statistical classifier, which was proposed by R.A. Fisher in 1936 [105]. LDA uses a simple discriminant function to classify instances of the sample. Its essence consists in maximizing the between classes sum of squares (SSbetween) and minimizing the within class SS (SSwithin) [106]. RDA ensures better performance (at least in certain cases in which the LDA and quadratic discriminant analysis fail) by using two regularization parameters in the discriminant function [107]. We have used the algorithm as implemented in the R package “klaR” [108].

Artificial neural networks (ANNs) are computational tools used in the prediction of continuous variables or in classification, inspired by the functioning of neurons in the human brain [109]. W.S. McCulloch and W. Pitts were the first, in 1943, to describe an algorithmic neuron, which is today known by their names (the McCulloch and Pitts neuron). Several years later, F. Rosenblatt described another algorithm inspired by the neuron functioning, the perceptron, which automatically “learns” optimal weighting coefficients, which are multiplied by the input variables to decide on the emission (or not) of an output by the neuron [110]. ANNs are particularly apt in modeling complex and non-linear relationships, as are often those found in chemistry, and thus they are seen as perfectly suitable for QSAR modeling [111]. We have used neural networks in three different R implementations: “neuralnet” [112], “nnet” [113], and “deepnet” [114].

The k nearest neighbor (kNN) algorithm is one of the simplest and efficient classification algorithms, being based on the idea of assigning an unknown sample to the class to which belong the k most similar compounds (k nearest neighbors). The similarity is assessed through the distance between each data point of the training sample and the unknown sample [115]. It has often been used successfully in QSAR applications [116,117]. We have used two R implementations of the algorithm: kknn [118] and the RWeka version (IBk) [104]. k values between one and 30 were used in the inner loop of the nested cross-validation.

For all classification algorithms hyperparameter tuning was performed within the inner loop of the nested cross-validation code, using random searches within specified bounds.

### 4.5. Performance Evaluation

To assess the performance of the models used in the study, we applied nested cross-validation with five folds in the inner loop and 10 folds in the outer one; in the case of BART models, which took a long time to compute, we used five folds for both loops. Cross-validation has a number of strengths that makes it superior to hold-out external validation [119], and nested cross-validation (also known as double cross-validation) is superior to the conventional, simple cross-validation, extending the concept of external validation to the whole dataset [120]. Nested cross-validation is, therefore, considered the state-of-the art approach for the validation of computational models such as QSAR [119], although to date only a very small number of published studies have used it (e.g., using “nested cross validation QSAR” as keywords in Medline returns only seven papers).

The following metrics were computed and assessed by nested cross-validation: balanced accuracy (BA), mean misclassification error (MMCE), sensitivity (true positive rate (TPR)), specificity (true negative rate (TNR)), positive predictive value (PPV), and Area Under the Receiver Operating Characteristics Curve (AUC) with their widely known definitions and equations [121,122]. We were interested in predicting whether a particular substance may induce DILI; therefore, we focused on increasing the balanced accuracy, as well as PPV. For this reason, we selected only those models that had both PPV and BA higher than 70%.

*Y-Randomization Test*. To examine to what extent models are the result of mere chance we applied a typical y-scrambling test [123] by permuting the toxicity concern label of the drugs from the dataset and re-developing the models following the same procedure as the one used for the selected models. We applied the process ten times, each time with different permutations and a different classification algorithm. The models thus built were evaluated for their performance using the same metrics as for the ones selected. If the models selected for use do not deliver a mere gambling result, the performance of the models built with the permuted data should be (considerably) worse than that of the performance of the selected models.

### 4.6. Virtual Screening

In order to explore the way in which the models perform on real-world data, we assembled the 79 selected QSAR models and applied them to predict the DILI potential of a dataset of 104, 619 compounds of the ZINC database (all compounds having names in the said database [124]). The 79 models were stacked in three different ways:

(a) By a majority vote applied to the classification performed by each model;
(b) By computing the average of the probabilities outputted by each model and then applying the 50% threshold to classify the compound as being of concern or of no concern (only 72 models outputted probabilities, 6 only made binary predictions);
(c) By developing meta-models using the predictions of the best 50 models (selected with the help of the same selection algorithms as for the building of the individual models) as independent variables for the final classification. We evaluated models based exclusively on the 50 best-performing individual models. We also built models that additionally included the dose and duration of treatment as supplementary features for the improvement of the performance.

### 4.7. Outliers and Applicability Domain

For the detection and analysis of outliers in the DILIrank dataset, we applied the isolation forest (IFOREST) and subspace outlier detection (SOD) algorithms, as implemented in the “solitude” [125] and “HighDimOut” R packages [126]. The applicability domain was assessed by three different methods, as detailed elsewhere [127]. The three methods are: (a) the three-stage heuristic procedure proposed by F. Sahigara et al. (2013), which uses individual decision thresholds for each new observation; (b) the Influenced Outlierness (INFLO) algorithm, which compares the local density of an observation with the mean density of its neighboring observations [30]; and (c) the Connectivity-based Outlier Factor (COF) algorithm, which is based on “chaining distances” and the distinction between “low density” and “isolativity” [31]. 

## Figures and Tables

**Table 1:** The most important molecular descriptors associated with drug-induced liver injury (DILI) by the 17 feature selection algorithms used.

Descriptor	Interpretation	Descriptor Block (group)	Frequency Occurring Among the First 5 Most Important Features	Sense of the Contribution *
Mp	mean atomic polarizability (scaled on Carbon atom)	Constitutional indices	12 (70.59%)	+
H%	percentage of H atoms	Constitutional indices	12 (70.59%)	−
GATS1m	Geary autocorrelation of lag 1 weighted by mass	2D autocorrelations	12 (70.59%)	−
SpPosA_B(m)	normalized spectral positive sum from Burden matrix weighted by mass	2D matrix-based descriptors	10 (58.82%)	+
MLOGP	Moriguchi octanol-water partition coeff. (logP)	Molecular properties	4 (23.53%)	+
PCR	ratio of multiple path count over path count	Walk and path counts	3 (17.65%)	+
totalcharge	total charge	Constitutional indices	2 (11.76%)	−
SM1_Dz.m.	spectral moment of order 1 from Barysz matrix weighted by mass	2D matrix-based descriptors	2 (11.76%)	+
SIC1	Structural Information Content index (neighborhood symmetry of 1-order)	Information indices	2 (11.76%)	+

* higher values associate with hepatotoxicity (+); higher values associate with lack of hepatotoxicity (−).

## Supplementary Materials

Supplementary materials can be found at https://www.mdpi.com/1422-0067/21/6/2114/s1.

## Abbreviations

AD	Applicability Domain
ANNs	Artificial Neural Networks
AUC	Area Under the Receiver Operating Characteristics Curve
BA	Balanced Accuracy
BART	Bayesian Additive Regression Trees
CV	Cross-Validation
DC-SVM	Divide-and-Conquer SVM
DILI	Drug induced liver injury
ECHA	European Chemicals Agency
ERT	Extremely Randomized Trees
FPR	False Positive Rate
IBk	an RWeka implementation of the knn algorithm
IFOREST	Isolation Forest
knn	k-nearest neighbours
LDA	Linear Discriminant Analysis
MMCE	Mean MisClassification Error
NIDDK	National Institute of Diabetes and Digestive and Kidney Diseases
OECD	The Organisation for Economic Co-operation and Development
PPV	Positive Predictive Value
QSAR	Quantitative Structure–Activity Relationship
RDA	Regularized Discriminant Analysis
RF	Random Forests
SOD	Subspace Outlier Detection
SVM	Support Vector Machines
TNR	True Negative Rate
TPR	True Positive Rate
