# Supplementary material for: Computational Models Using Multiple Machine Learning Algorithms for Predicting Drug Hepatotoxicity with the DILIrank Dataset
Source: Int J Mol Sci. 2020 Mar 19;21(6):2114. doi: 10.3390/ijms21062114 (PMC7139829; doi:10.3390/ijms21062114)
Supplement: Supplementary file 1 [file ijms-21-02114-s001.zip › Table S3.docx]

Table S3. Blocks of molecular descriptors computed on the DILIrank data set

| **No.** | **Descriptor block** | **Number of descriptors** |
| --- | --- | --- |
| 1 | Constitutional descriptors | 47 |
| 2 | Ring descriptors | 32 |
| 3 | Topological indices | 75 |
| 4 | Walk and path counts | 46 |
| 5 | Connectivity indices | 37 |
| 6 | Information indices | 50 |
| 7 | 2D matrix-based descriptors | 607 |
| 8 | 2D-autocorrelations | 213 |
| 9 | Burden eigenvalues | 96 |
| 10 | P-VSA-like descriptors | 55 |
| 11 | ETA indices | 23 |
| 12 | Edge adjacency indices | 324 |
| 13 | Functional groups count | 153 |
| 14 | Atom-centred fragments | 115 |
| 15 | Atom-type E-state indices | 172 |
| 16 | CATS 2D | 150 |
| 17 | 2D atom pairs | 1596 |
| 18 | Molecular properties | 20 |
| 19 | Drug-like indices | 28 |
