# Supplementary figures and images for: Computational Models Using Multiple Machine Learning Algorithms for Predicting Drug Hepatotoxicity with the DILIrank Dataset
Source: Int J Mol Sci. 2020 Mar 19;21(6):2114. doi: 10.3390/ijms21062114 (PMC7139829; doi:10.3390/ijms21062114)

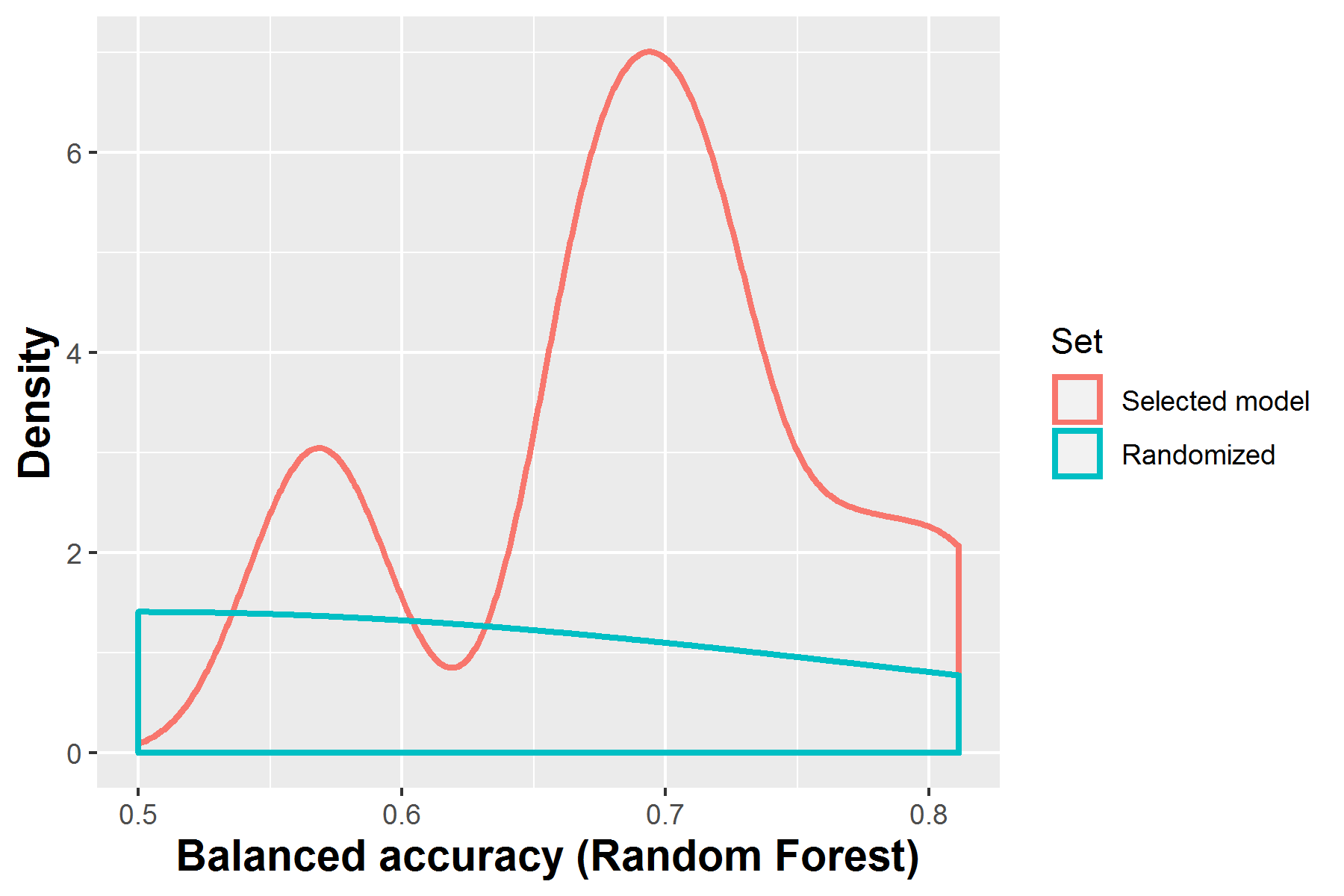

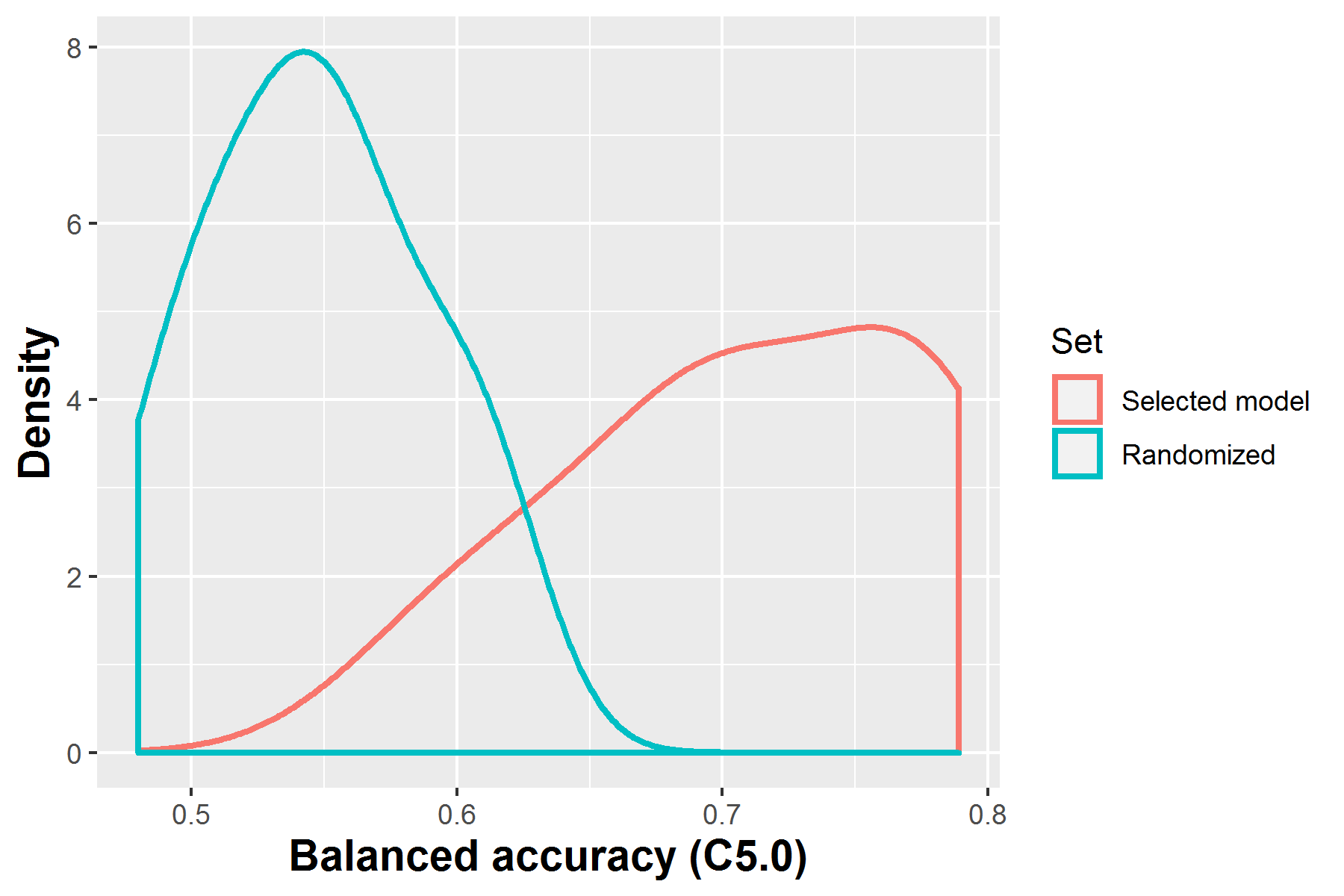

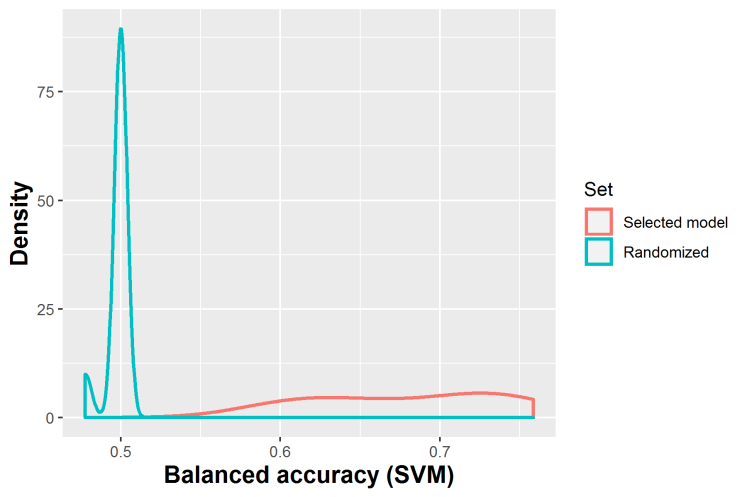

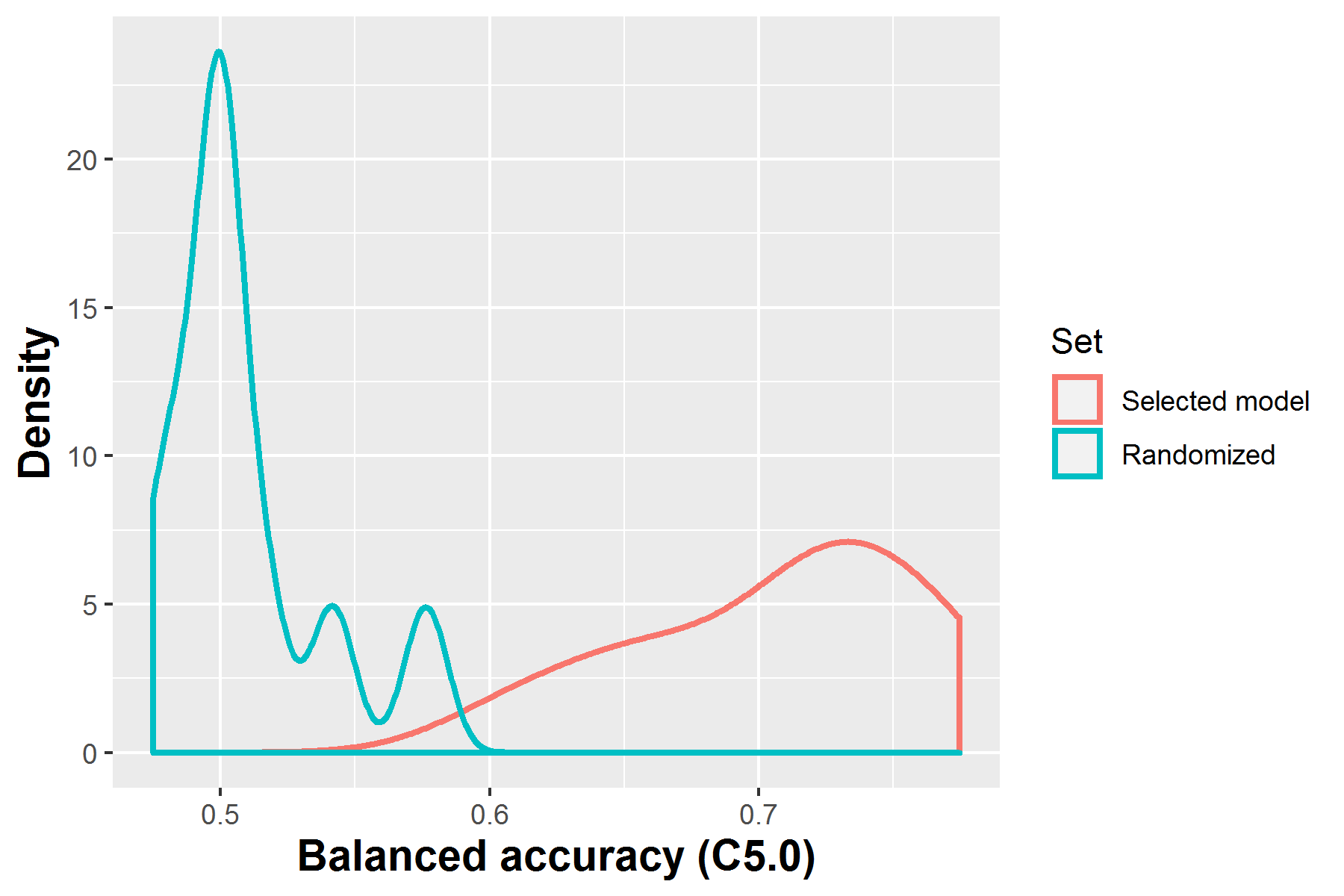

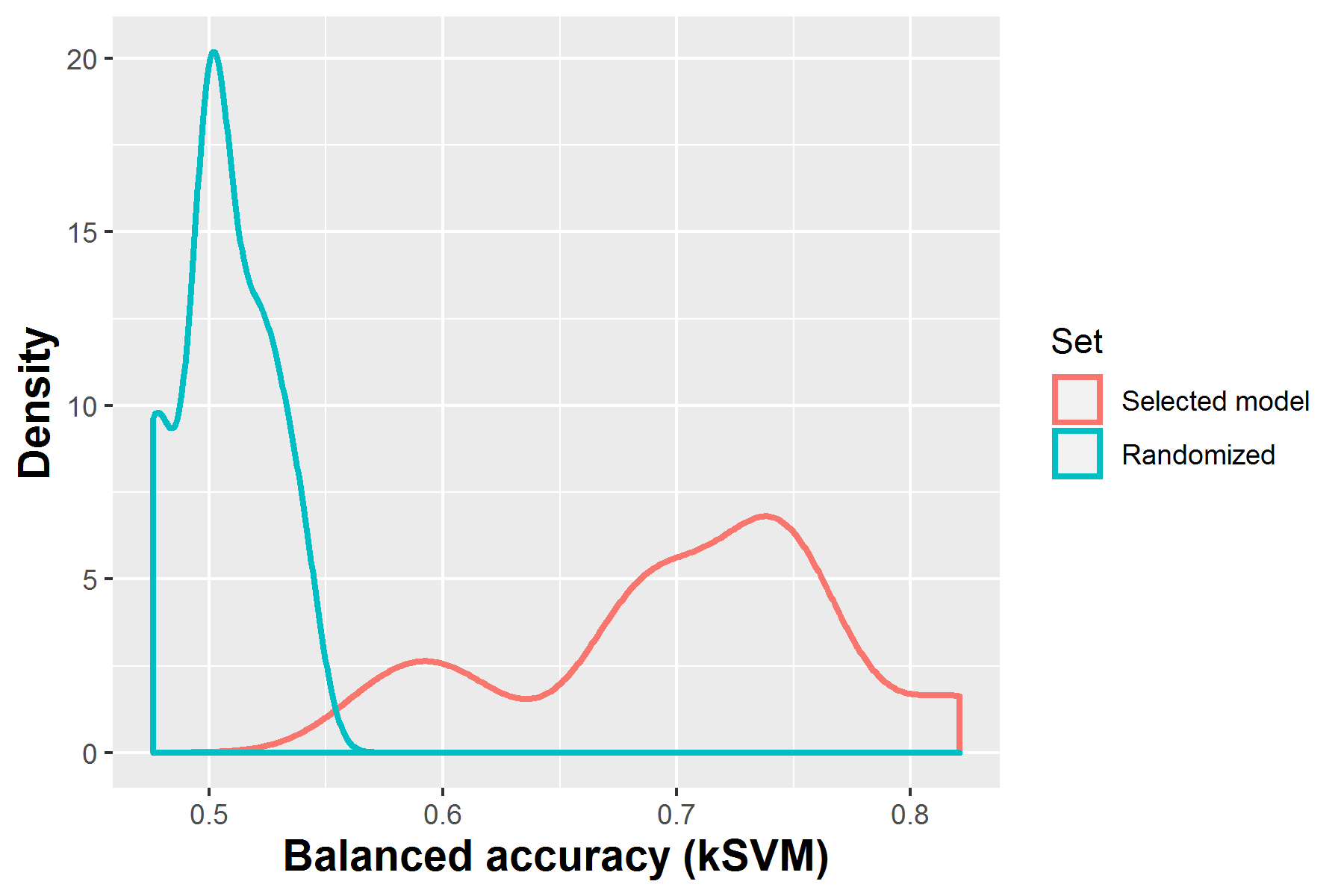

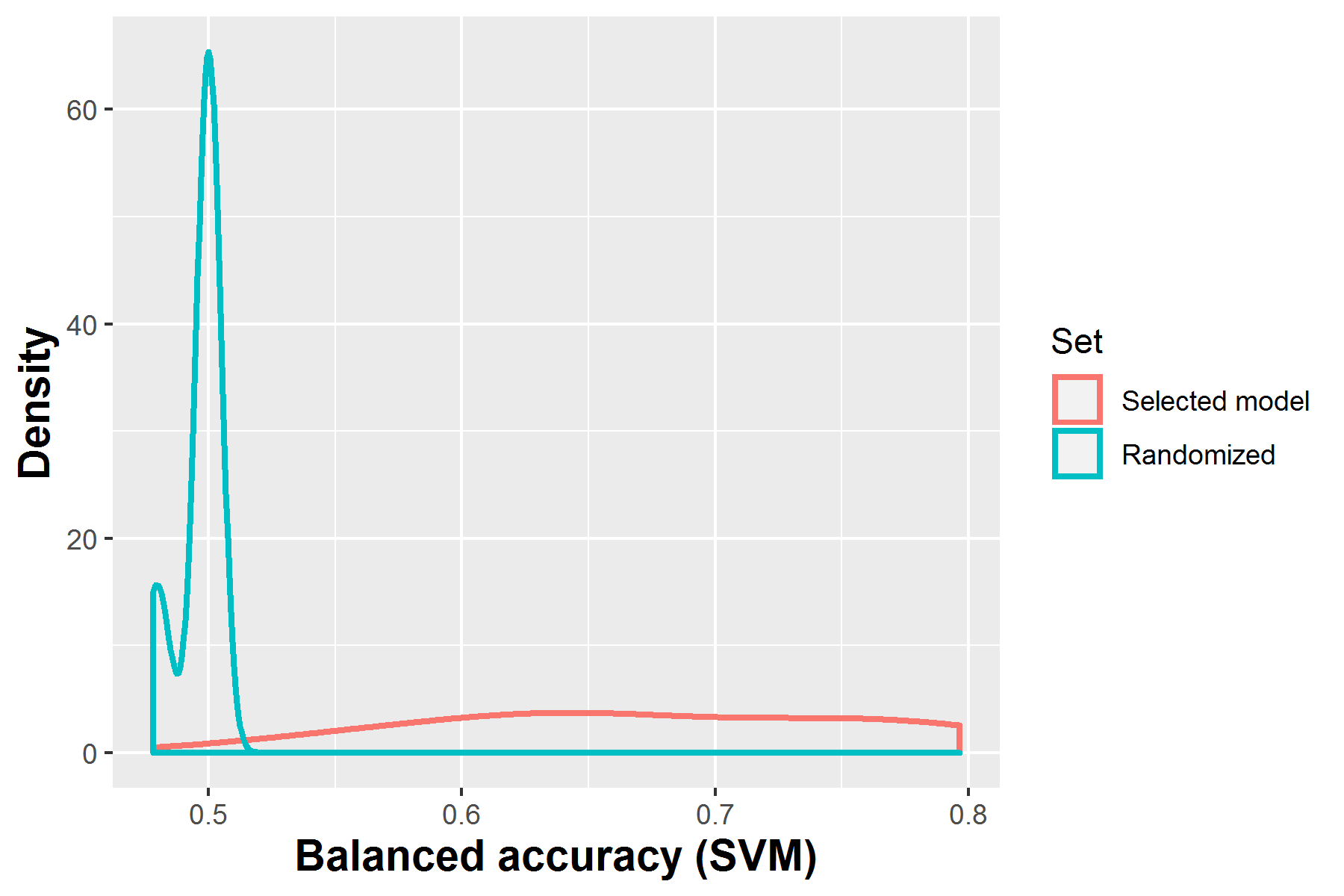

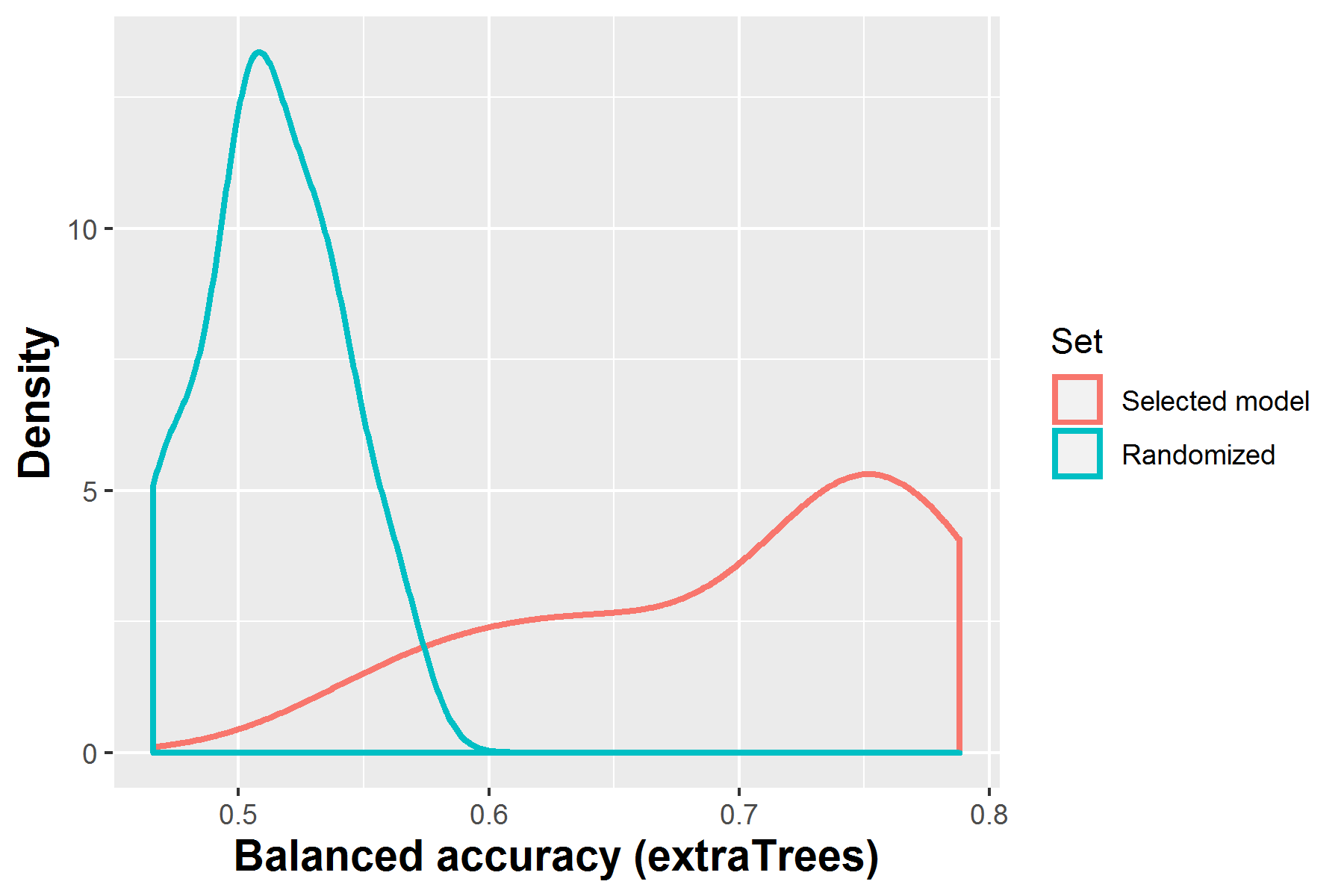

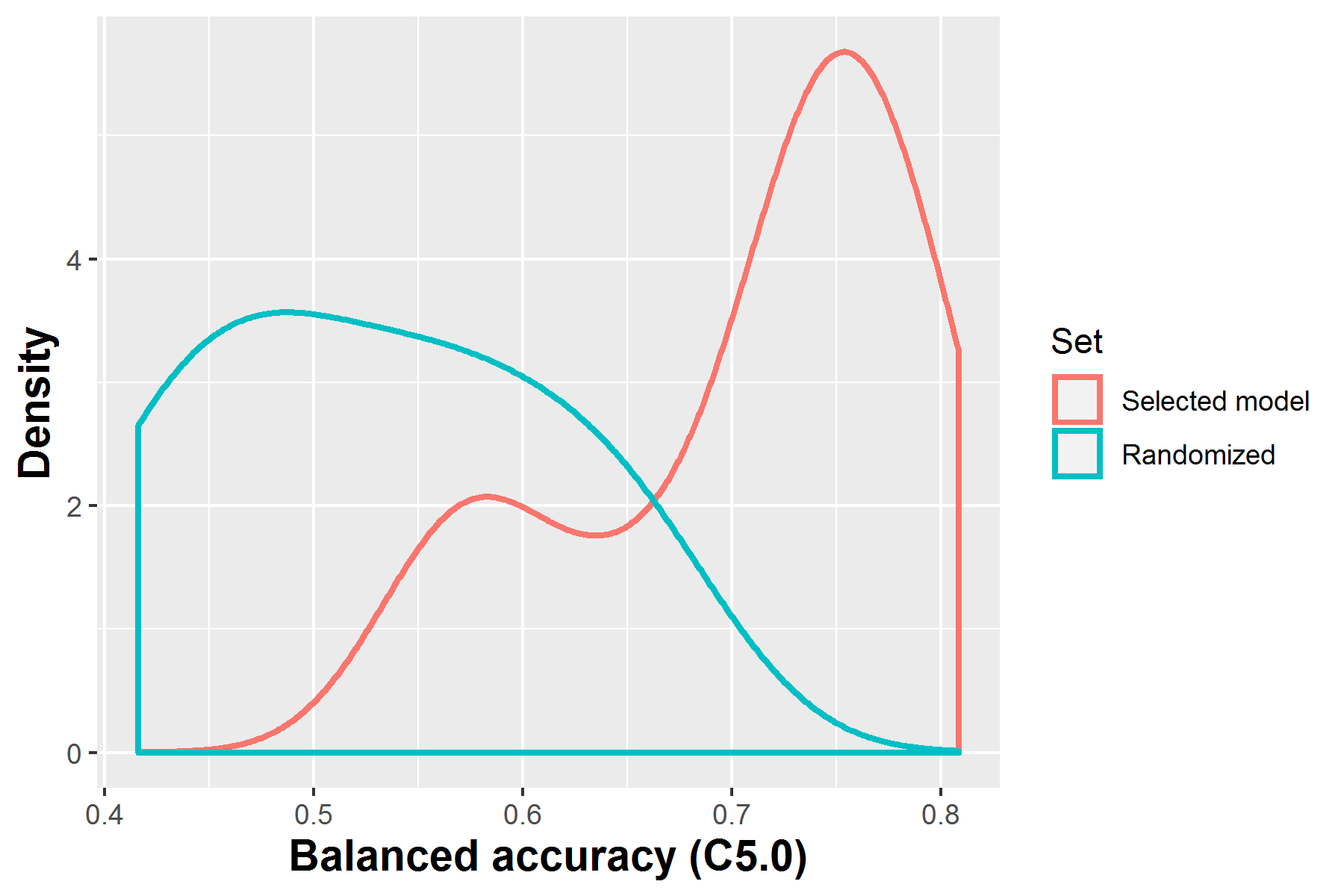

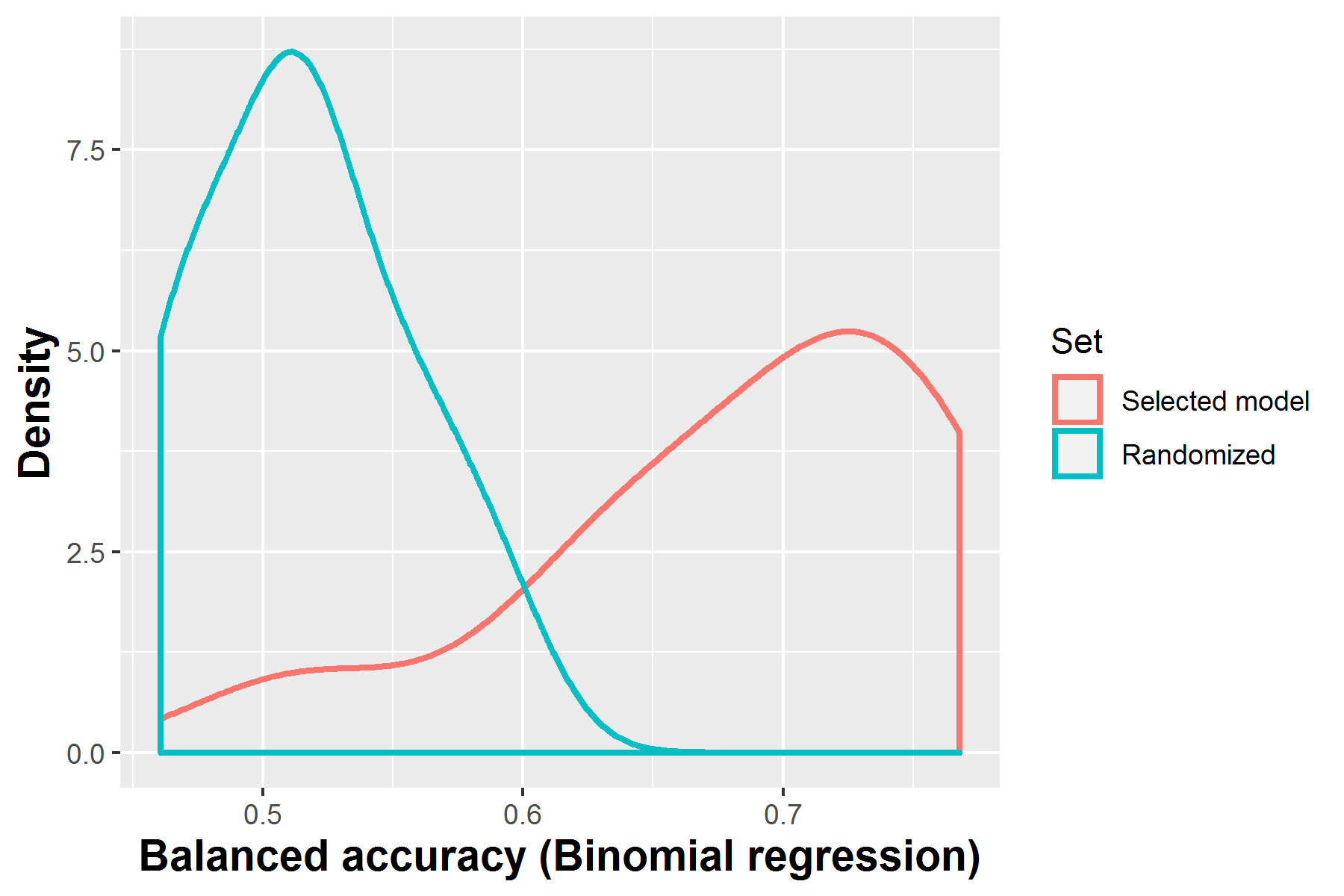

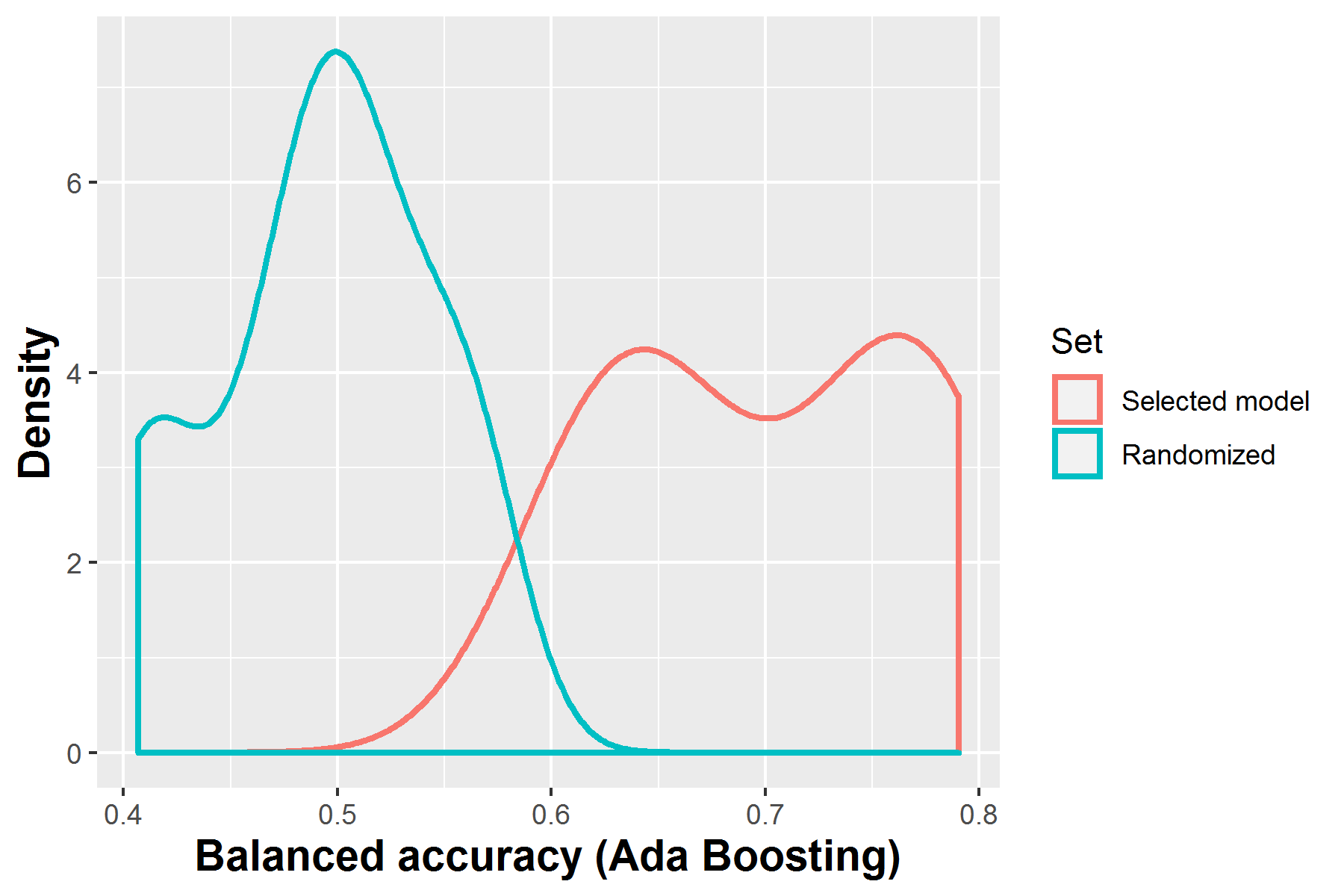

Supplement: Supplementary file 1 [file ijms-21-02114-s001.zip › Figure S4.docx]

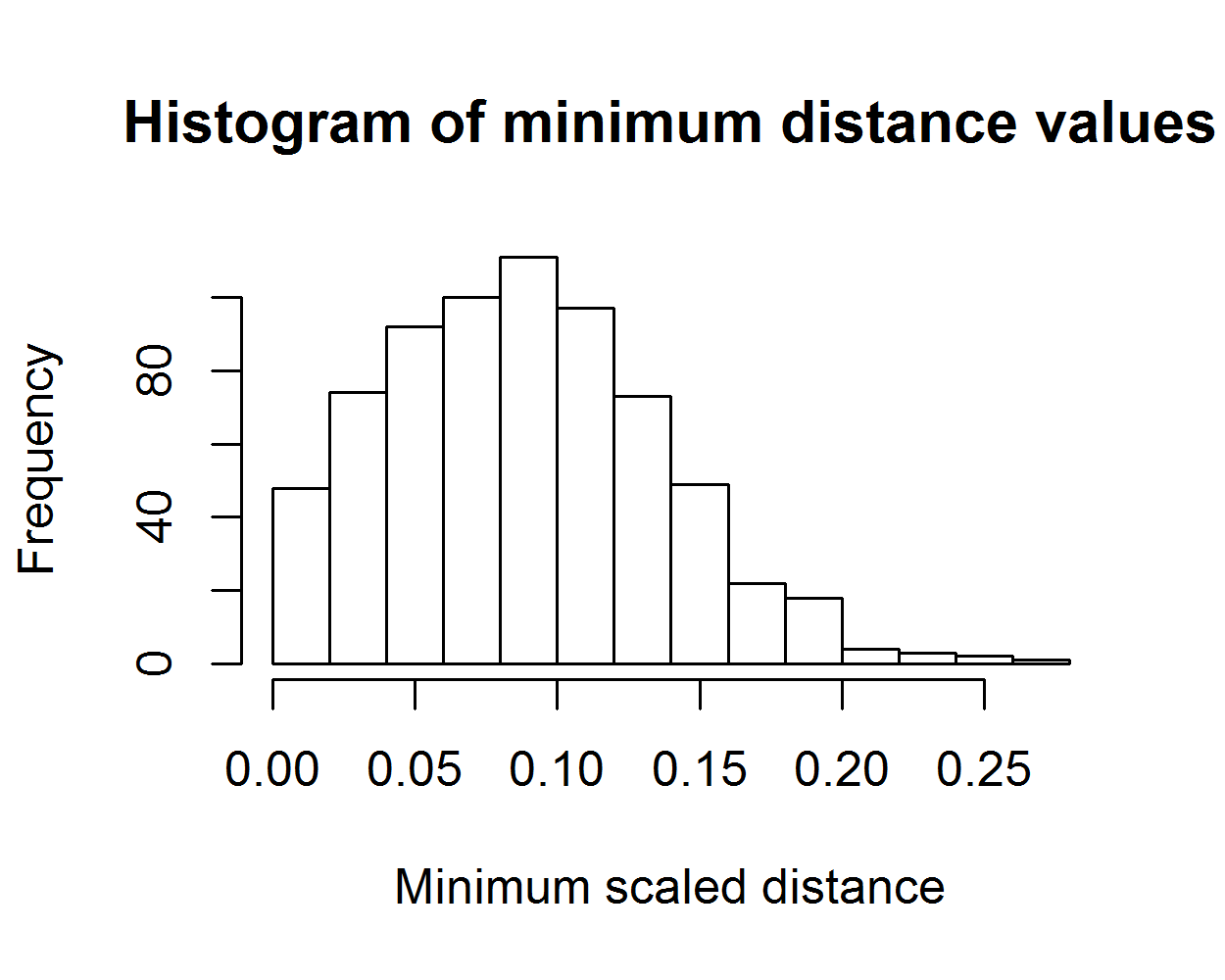

Supplement: Supplementary file 1 [file ijms-21-02114-s001.zip › S1Fig_minimum_distance_values.tif]

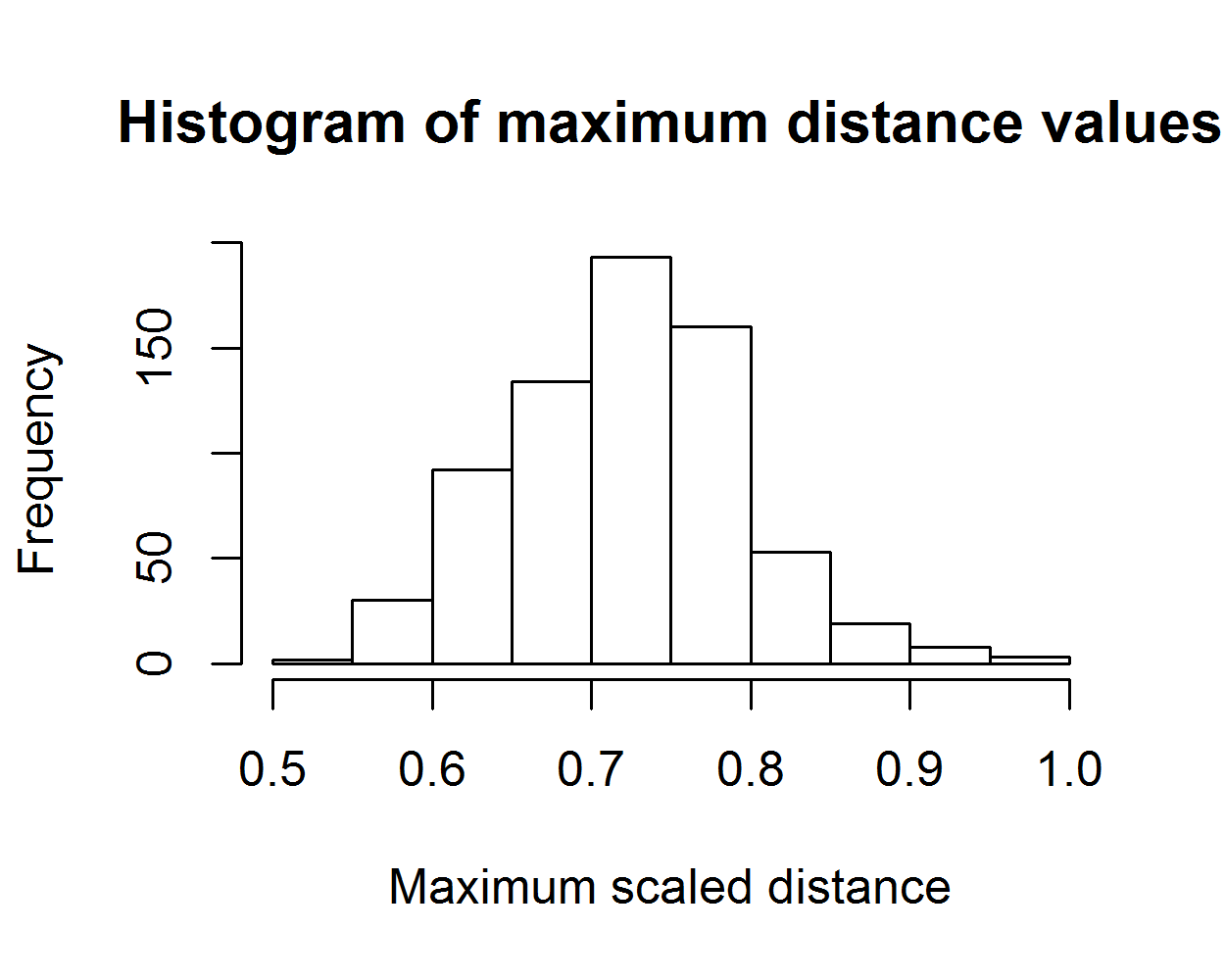

Supplement: Supplementary file 1 [file ijms-21-02114-s001.zip › S2Fig_maximum_distance_values.tif]

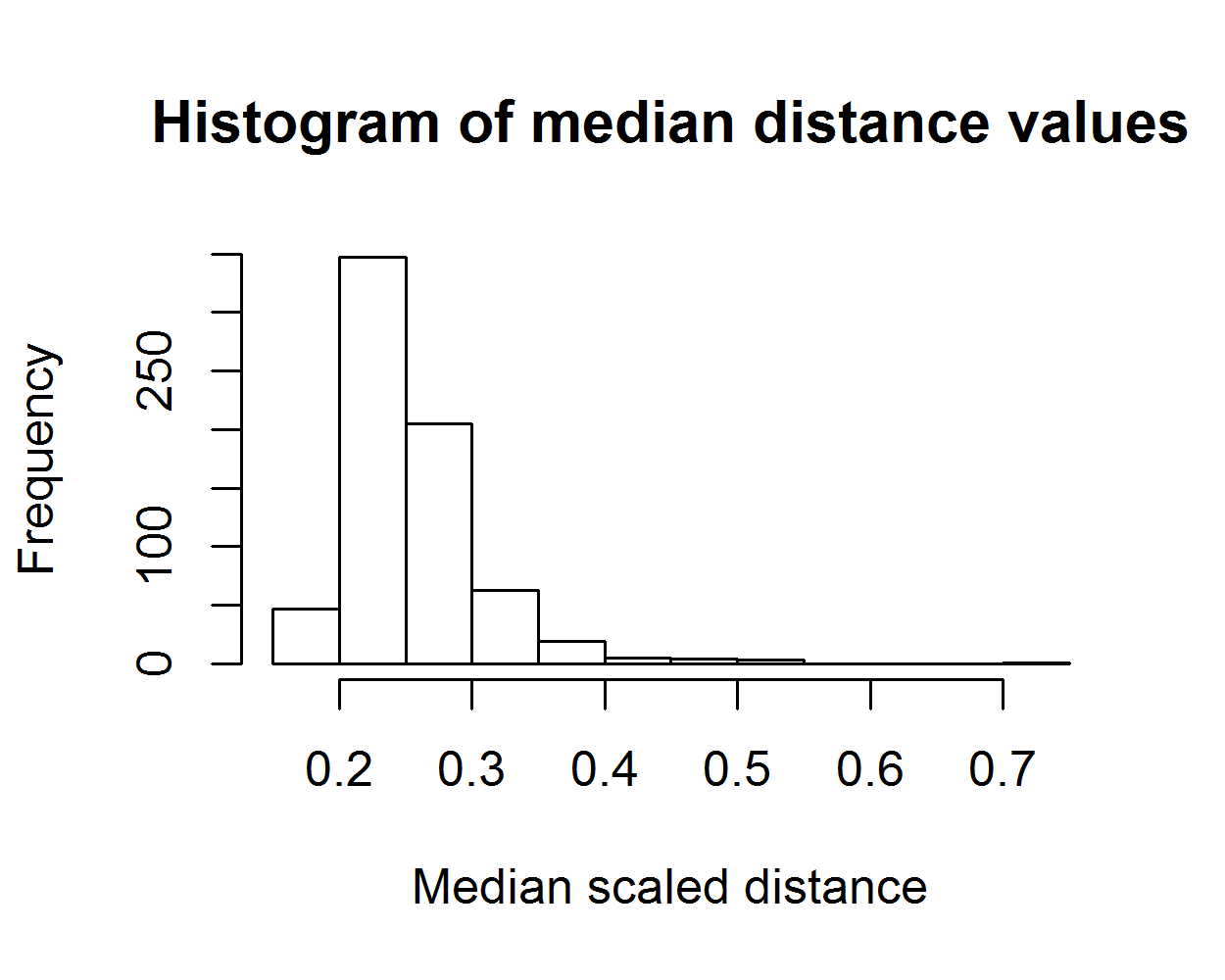

Supplement: Supplementary file 1 [file ijms-21-02114-s001.zip › S3Fig_median_distance_values.tif]
